# Balancing Privacy and Utility in Secondary Data Use to Inform Policy

**DOI:** 10.23889/ijpds.v1i1.357

**Published:** 2017-04-19

**Authors:** Xinjie Cui, Robyn Blackadar

**Affiliations:** 1 Alberta Cebtre for Child Family and Community Research

## Objectives

Using existing data for research can generate new knowledge and evidence for policy with relatively little cost. Privacy concerns are paramount in such secondary usage of data collected on human subjects. Information privacy protection and data security are critical considerations in reuse and repurposing of data especially linked data, longitudinal data, and large amounts of data. Data sharing and privacy protection are both in the public interest and we need to assess the risk of “doing” (sharing) as well as the risk of "not doing" (not sharing or not protecting). 

## Approach

The Alberta Centre for Child Family and Community Research (the Centre) establishes the Child Youth Data Lab that links and analyzes administrative data from multiple provincial ministries and the Child Data Centre of Alberta that repurposes research data and manages its access for reuse. The Centre partners with provincial Office of the Information Privacy Commissioner, Research Ethics Boards and leaders in the research communities and technology industry to design and develop measures to enable secondary use while safeguarding the data, and to explore and adopt best practices on data sharing processes, governance, and technologies. 

## Results

In principle current privacy laws and regulations provide good guidance in collection, use, and disclosure of data, however there is a lack of consistency in the interpretation of these laws at the operational level with regard to secondary data use. The experiences of establishing different data sharing models at the Centre through multiple initiatives are discussed. Cross-sectoral broad partnership brings understanding and builds trusting relationships, which are crucial to establishing data sharing processes. The recognition of the significance of secondary data use to provide direction for policy and program development at the executive level provides commitment for data sharing initiatives. Strong governance structure consists multi-level ministry and multiple stakeholder involvement ensures ongoing support and engagement. The highest data security standards and anonymous solution for data linkage enables the sharing of data with good privacy protection.

## Conclusions

Secondary use of data to improve system performance and contributing to scientific discovery has been broadly recognized. A balance between utility and privacy can be realized through broad partnership in building proper governance, technology, processes and policies.

